# RETRACTION: Identification of Ferroptosis-Related Gene Prognostic Signature and HSF1 for Reversing Doxorubicin and Gemcitabine Resistance in Uterine Carcinosarcoma

**DOI:** 10.1155/dim/9762860

**Published:** 2025-07-21

**Authors:** Disease Markers

RETRACTION: S. Han, Q. Liu, Z. Yang, et al., “Identification of Ferroptosis-Related Gene Prognostic Signature and HSF1 for Reversing Doxorubicin and Gemcitabine Resistance in Uterine Carcinosarcoma,” *Disease Markers* 2022, no. 1 (2022): 1–16, https://doi.org/10.1155/2022/6400227.

The above article, published online on 12 January 2022 in Wiley Online Library (wileyonlinelibrary.com), has been retracted by John Wiley & Sons Ltd.

The retraction has been agreed following communication received from the authors indicating that they consider the contents of the article to be unreliable, in part due to the duplication of panels in Figure 5G (MES-SA Doxorubicin and FU-MMT-1 Sh#2). A subsequent investigation has additionally identified concerns with the peer review process.

